# Mental compromise in SARS-CoV-2 infected patients is multicausal, organic or inorganic

**DOI:** 10.1093/braincomms/fcab218

**Published:** 2021-09-22

**Authors:** Josef Finsterer, Fulvio A Scorza

**Affiliations:** 1 Klinik Landstrasse, Messerli Institute, Vienna, Austria; 2 Disciplina de Neurociência. Universidade Federal de São Paulo/Escola Paulista de Medicina (UNIFESP/EPM), São Paulo—SP 04039-00, Brazil

##  

With interest, we read the review article by Meier et al. about the neurological and mental health consequences of COVID-19 [1]. It was concluded that future medication for COVID-19 may keep labour force healthy and may be also beneficial for Alzheimer’s disease or other types of dementia [1]. The review is appealing but raises concerns and comments.

Mental complications of SARS-CoV-2 infections may either originate from the impact of the infection on the individual or collective psycho-social status causing uncertainty, fear, mood disorder or psychosis, or from neurological disease as a complication of the viral infection. It is well established that SARS-CoV-2 may not only affect the lungs but generally all organs/tissues, including the central nervous system [2]. Central nervous system disease triggered by the virus includes viral or immune encephalitis, myelitis, acute, haemorrhagic, necrotizing encephalitis, diffuse leukoencephalopathy, acute disseminated encephalomyelitis, ischaemic stroke, intracerebral bleeding, subarachnoid bleeding, venous sinus thrombosis, posterior reversible encephalopathy syndrome, cerebral vasculitis, cerebral vasoconstriction syndrome or neuroleptic malignant syndrome [2]. Most of these conditions can go along with psychiatric or mental abnormalities.

Not addressed in the review, for example, were viral and immune encephalitis, both having been reported as complications of SARS-CoV-2 infections [3]. Encephalitis may manifest as abnormal behaviour or delirium [3]. Patients with SARS-CoV-2 associated encephalitis may also present with confusion in addition to focal neurological deficits [4]. Single patients with encephalitis and mania have been reported. Such patients may favourably respond to steroids or intravenous immunoglobulins, not only with regard to encephalitis but also with regard to the mental condition.

Since mental disease in patients with SARS-CoV-2 infections may have a neurological origin, it is crucial that patients with mental compromise are thoroughly investigated for neurological causes of the psychological compromise. Work-up for neurological disease should not only include a profound individual and family history and a clinical neurologic exam but also imaging of the central nervous system and the vascular supply with contrast medium, EEG, and eventually investigations of the cerebro-spinal fluid. Assuming that SARS-CoV-2 infections are associated with neuronal or glial death, it is recommended that COVID-19 patients undergo appropriate neuropsychological testing on admission and on dismissal from the hospital.

An issue not addressed in the review is that the anti-COVID-19 medication may be neurotoxic and may be responsible for long-term mental impairment in patients experiencing a SARS-CoV-2 infection. Particularly, from chloroquine it is known that it can cause severe psychiatric disease and some patients may even commit suicide [5]. In a single patient with COVID-19 the loading dose of favipiravir triggered an acute psychosis [6]. In a study on the side effects of tocilizumab given prior to haematopoetic stem cell transplantation, the prevalence of depression was significantly increased compared to controls [7]. From steroids, it is well-known that they may induce a drive increase, hallucinations, or even psychosis. From linezolid, it is known that it can cause serotonin syndrome [8].

As virus RNA has been repeatedly documented in neurons, glial cells, or the cerebro-spinal fluid [9], pathways must exist along which the virus enters the brain. In this sense, SARS-CoV-2 may not only enter the brain via retrograde migration through axons of cranial nerves or the blood stream and directly crossing the BBB but also by incorporation into lymphocytes, which may achieve the ability to cross the blood–brain barrier together with the virus [10].

Assuming that SARS-CoV-2 causes chronic cerebral inflammation, one would expect pleocytosis, increased cerebro-spinal fluid protein, or positive oligoclonal bands. However, cerebro-spinal fluid abnormalities of this kind have been only reported in patients with neurological involvement in COVID-19.

We do not agree with the notion that mental health compromise in SARS-CoV-2 infected patients is due to cerebral hypoxia. Patients with severe COVID-19 require oxygen supplementation or even mechanical ventilation. These patients are usually carefully monitored for sufficient oxygenation on an ICU and are not exposed to hypoxia. A further argument against hypoxia as the cause of mental disturbance is that patients with severe COVID-19 usually do not present with the classical stigmata of hypoxia on cerebral imaging.

Hyposmia/anosmia and hypogeusia/ageusia may not only result from invasion of neurons but simply from the occupation of the taste buds of the tongue or the pharynx or the olfactory cells in the nasal epithelium by the virus. Concerning ‘lymphohistiocytosis’ it should be considered that COVID-19 patients may also experience lymphopenia.

Overall, the review has several limitations which challenge the results and their interpretation. SARS-CoV-2 infected patients developing compromise of mental health require clinical and instrumental neurological work-up. Before attributing mental dysfunction to psychiatric involvement in the infection, central nervous system involvement with psychiatric presentation must be thoroughly excluded. To assess if mental health compromise has truly an organic background the brain needs to be extensively investigated. Central nervous system compromise from SARS-CoV-2 may be at variance from the pathophysiology in HIV encephalopathy or in Alzheimer’s disease. Science should prevent hysteria from becoming more harmful than the virus.

## Competing interests

The authors report no competing interests. 
